# Access to digital and social media among Romanian HIV/AIDS clinical providers

**DOI:** 10.1080/16549716.2018.1513445

**Published:** 2018-09-06

**Authors:** Carmen Manciuc, Brooke A. Levandowski, Erin Muir, Amanda Radulescu, Monica Barbosu, Timothy D. Dye

**Affiliations:** a Department of Infectious Disease, Grigore T. Popa, University of Medicine and Pharmacy, Iași, Romania; b Department of Obstetrics and Gynecology, University of Rochester School of Medicine and Dentistry, Rochester, NY, USA; c Department of Epidemiology, Iuliu Hatieganu University of Medicine and Pharmacy, Cluj-Napoca, Romania

**Keywords:** Romania, social media, internet, HIV, AIDS

## Abstract

The WHO/UNAIDS suggests that digital tools – such as social media and online training opportunities, can connect providers in difficult social and medical contexts to providers elsewhere for guidance, support, and advice. Social media is emerging as an innovative option for connecting clinicians together and for enhancing access to professional resources. In Romania, characterized by an atypical HIV/AIDS epidemic which is further challenged by a range of access complexities, it is unclear how often – and which kinds of – social media clinicians use to support clinical care. This study was conducted to ascertain social media use for clinical providers based in two regions of Romania (Transylvania and Moldavia) who face distance challenges that could potentially be alleviated by social media interaction. We used an online survey to understand what social media are currently popular and perceived to be useful for learning clinical information. Descriptive and bivariate analyses were conducted. Providers indicated Facebook and WhatsApp were the most common social media platforms, with 62% and 45% reporting daily use, respectively. Providers who used one media platform were significantly more likely to use another social media platform (p < .05). These data are helpful for creating an online training platform on HIV/AIDS for Romanian clinical providers.

## Background

Online information on the internet is omnipresent and social media is pervasive, allowing unlimited professional networking, sharing of opinions, professional education and training, and dissemination of knowledge [–3]. Medical professionals are using the variety of social media outlets to improve patient-provider communication, supplement their professional development, provide and receive peer support through networking capabilities, and contribute to public health prevention and service [2,4]. Indeed, greater than 90% of physicians reported using social media outlets; regular use of Facebook accounts by physicians was estimated to have grown from 13% to 47% in 2011 [4,5].

UNAIDS has established that ‘universal access to HIV prevention, treatment, care and support services to be as ubiquitous as mobile phone coverage,’ and that the way to achieve this goal is by ‘harnessing technology’ like using mobile health interventions [6]. Already this goal is being realized through information and communication technologies (ICTs) like the internet and social media that are used in many health scenarios, including ending the HIV/AIDS epidemic [7–12]. Consequently, the internet and social media are valuable tools for targeting people at risk of HIV/AIDS, enhancing support and care for people living with HIV/AIDS, and training clinical providers to better care for their HIV/AIDS patients. Romania – with very strong and widespread digital technologies and increasing connectivity – could experience substantial improvement in their AIDS epidemic with the adoption of ICTs [7,13].

Identifying the social media outlets used by the target audience is a critical first step to ensuring the information flows directly to consumers and is subsequently utilized to address public health concerns [8,14]. Therefore, the purpose of this study was to determine how Romanian clinical providers interact with specific social media platforms to better understand the potential of the current ICT infrastructure, specifically social media and other online sites used to further clinical education, and to improve HIV-related care. As online information exchanges, social support groups, and training modules have been effective in other contexts [9,11,12,15–17], this study will determine the current social media practices specifically in Romania as a foundation of future collaborative construction of a new digital platform.

## Methods

An online survey was conducted among Romanian HIV/AIDS clinical providers to determine their existing and upcoming continuing medical education and capacity-building needs surrounding HIV/AIDS. Romanian HIV/AIDS clinical providers were recruited using snowball sampling and referral from the Cluj-Napoca and Iaşi HIV clinical hubs in the Transylvania and Moldavia regions of Romania, respectively. Two authors (CM and AR) sent clinical providers in their professional networks an email with a link to an online survey in REDCap, which was open for 12 days in July 2017[18] Email recipients were encouraged to forward the link to their own professional networks of HIV/AIDS clinical providers.

The needs assessment survey was based on the New York State AIDS Institute Clinical Education Initiative’s domestic assessment of HIV/AIDS training needs, updated to reflect the Romanian context. Participants were asked to indicate the frequency of their use of media sources on a 5 point Likert scale (never, rarely, several times per month, several times per week, daily). Social and electronic media sources included Facebook, Instagram, LinkedIn, WhatsApp, Skype, and other. Sparse demographic variables were collected, such as primary occupation, practice setting, urban or rural location, and geographic regions of Transylvania and Moldavia. Descriptive statistics were used to assess frequencies. Bivariate analyses were conducted using two-way ANOVAs, two-tailed Pearson Correlations, and Pearson chi-square tests to ascertain relationships between variables, testing each of the demographic variables (primary occupation, practice setting, urban/rural, and Transylvania/Moldavia) against each of the social media platforms. Statistics were calculated using SPSS 24.0 [19].

## Results

**Figure 1.**
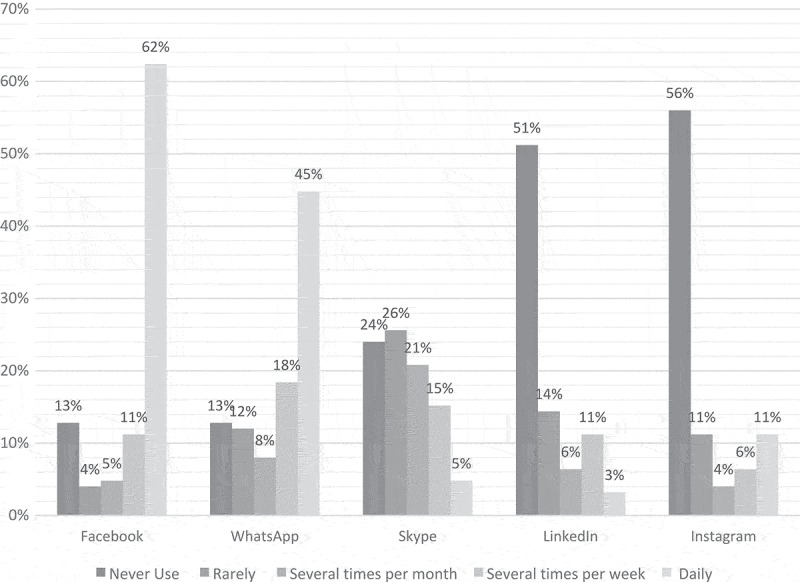
Frequency of social media platform use among Romanian HIV/AIDS clinical providers (n = 125), 2017.

One hundred twenty-five Romanian clinical providers completed the online survey. Of those, 82% were physicians, 74% worked in hospitals, and 94% were in urban settings, with 63% located in Transylvania and 37% in Moldavia. Overall, Facebook and WhatsApp were the social media sites used most frequently, with 74% and 63% reporting daily or weekly use, respectively, followed by Skype (20% used daily or weekly). Skype was used more frequently than Instagram and LinkedIn (41%, 21% and 20% reporting monthly use, respectively) which were used rarely (p < .05). Over half of respondents reported never using Instagram and LinkedIn (Figure 1).

Bivariate analyses were conducted comparing social media platforms with the demographic characteristics described above, with no significant associations found. Bivariate analyses were conducted comparing social media platforms with the other social media platforms, finding that users of one social media platform were significantly more likely to use at least one other platform. Facebook users were significantly more likely to use Instagram (p = .009) and WhatsApp (p = .02). LinkedIn users were significantly more likely to use Instagram (p < .001), WhatsApp (p = .03) and Skype (p = .01).

Bivariate analyses were conducted comparing never using each social media type or daily use of each social media type to the sociodemographic characteristics described above (Table 1). The HIV/AIDS clinical workforce living in Transylvania and Moldavia differed in their use of social media: about one-fifth (18.7%) of Transylvanians never use Facebook, compared to only 4.5% of Moldavians (p = .02). Moldavians are more likely to use Facebook daily (79.5% vs. 57.3%, p = .01, respectively), but also more likely to use Instagram daily (22.5% vs 7%, p = .02, respectively). The HIV/AIDS clinical workforce who were physicians compared to other specialties differed in their use of Facebook as a platform: while Facebook is the most used social media platform in this study, 16.3% of physicians never use Facebook (compared to 0% of non-physicians, p = .04). That said, 64.3% of physicians use Facebook daily (compared to 71.4% of non-physicians, p = .4). Almost half (46.7%) of providers used WhatsApp daily, with non-physicians being significantly more likely to be daily users (66.7% vs. 42.4%, p = .04). Four percent of physicians reported daily use of LinkedIn, while 0% of non-physicians reported daily use (p = .55).

**Table 1.** Social Media Usage and Preference, HIV/AIDS Clinical Providers, Romania, 2017.

	Transylvania	Moldavia	MD	Not MD^a^	Hospital	Not Hospital^b^
Facebook						
Mean Usage^c^ (mean, SD)	2.9 (1.6)	3.6 (1.0)^d^	3.0 (1.5)	3.5 (1.0)	3.2 (1.5)	3.0 (1.4)
Daily Usage (%, n)	57.3 (43)	79.5 (35)^d^	64.3 (63)	71.4 (15)	67.8 (59)	59.4 (19)
Never Use (%, n)	18.7 (14)	4.5 (2)^d^	16.3 (16)	0.0 (0)^d^	13.8 (12)	12.5 (4)
WhatsApp						
Mean Usage^c^ (mean, SD)	2.8 (1.5)	2.6 (1.5)	2.7 (1.5)	3.1 (1.4)	2.8 (1.5)	2.7 (1.5)
Daily Usage (%, n)	50.6 (39)	39.5 (17)	42.4 (42)	66.7 (14)^d^	47.3 (43)	44.8 (13)
Never Use (%, n)	13.0 (10)	14.0 (6)	15.2 (15)	4.8 (1)	14.3 (13)	10.3 (3)
Skype						
Mean Usage^c^ (mean, SD)	1.4 (1.1)	1.6 (1.3)	1.5 (1.2)	1.2 (1.3)	1.6 (1.3)	1.1 (0.9)
Daily Usage (%, n)	2.8 (2)	9.8 (4)	4.2 (4)	11.1 (2)	7.0 (6)	0.0 (0)
Never Use (%, n)	26.4 (19)	26.8 (11)	24.2 (23)	38.9 (7)	25.6 (22)	29.6 (8)
LinkedIn						
Mean Usage^c^ (mean, SD)	0.7 (1.1)	1.2 (1.4)	0.92 (1.3)	0.40 (0.7)	0.9 (1.3)	0.7 (0.9)
Daily Usage (%, n)	1.4 (1)	7.7 (3)	4.3 (4)	0.0 (0)	4.9 (4)	0.0 (0)
Never Use (%, n)	65.2 (45)	48.7 (19)	57.0 (53)	73.3 (11)	62.2 (51)	50.0 (13)
Instagram						
Mean Usage^c^ (mean, SD)	0.8 (1.3)	1.2 (1.7)	0.87 (1.4)	1.3 (1.6)	0.94 (1.5)	0.93 (1.4)
Daily Usage (%, n)	7.0 (5)	22.5 (9)^d^	12.1 (11)	15.0 (3)	13.4 (11)	10.3 (3)
Never Use (%, n)	67.6 (48)	55.0 (22)	64.8 (59)	55.0 (11)	63.4 (52)	62.1 (18)

MD = medical doctor; SD = standard deviation

^a^ Primary professional occupations included pharmacists, nurses and social workers.

^b^ Primary practice settings included OBGYN clinics and private practices

^c^ Variable measured on a Likert scale where 0 = Never Use; 1 = Rarely; 2 = Monthly; 3 = Weekly; 4 = Daily;

^d^ *P *< .05

## Discussion

Social media is a standard component of contemporary communications, both inside and outside of the workplace. This needs assessment identifies critical information about reaching the Romanian HIV/AIDS workforce, engagement with whom is necessary to make progress on difficult and complex HIV-related problems in the country. Many studies have been conducted among physicians regarding their use of the internet to access such resources, which show social media use is commonly utilized to link physicians to a variety of online professional resources, such as clinical guidelines, peer-reviewed medical and resource journals, and other resources such as continuing medical education courses provided both through live and online training [2,3,14,20]. Social media benefits include the opportunity for real-time responses at a relatively low cost, information sharing, increased accessibility and peer support [2]. This research corroborates our findings, showing that Romanian physicians were using social media resources such as Facebook and WhatsApp daily, suggesting that these platforms can be used for connecting Romanian physicians to continuing medical education resources. As over two-thirds of non-physicians are using Facebook and WhatsApp daily, these platforms are a relatively easy way to connect this population to guidelines, journals and other resources on HIV prevention, treatment, and care.

Our findings showed that HIV/AIDS clinical providers in Moldavia were more likely to use Facebook and Instagram daily, compared to those in Transylvania. This was a surprising finding, as Transylvania is considered to be the most technologically advanced city in Romania, housing many national and international technology businesses. We hypothesize that Moldavian providers are more curious in exploring online resources for medical education, and note that this finding deserves further investigation.

This study is limited by the convenience sample of HIV/AIDS clinical providers who completed the survey, as it is not representative of all HIV/AIDS clinical providers. In addition, these findings may not represent HIV/AIDS clinical providers in other areas of Romania, including those in rural areas. Last, although the survey requested information on how clinical providers use social media for online education, respondents may have described their general use of social media, and not their specific professional use of social media.

This study identified that Facebook and WhatsApp are clearly highly used and offer great potential for reaching clinicians with engagement, training opportunities, clinical communication, and support. Providers can collaborate and gain social support using social media such as Facebook groups, WhatsApp chats, and Skype calls to exchange advice, ask questions, and address concerns [4,10]. Especially in Romania where HIV stigma is high, it could be beneficial to have an online resource to provide social support. A Facebook group could be easy to access and use, offering differing levels of anonymity to protect both clinicians and the patients they serve [20–23]. Such a group could provide information and emotional support to enhance provider knowledge, deconstruct stigmatic myths, and compare methods of patient care, including validation and encouragement of proper, effective, ethical methods.

## Conclusions

Social media offers important resources and opportunities that can help bridge the digital divide; extending international collaboration, engagement, and training to social media platforms can help provide needed access to training and communication opportunities that can help clinicians who are otherwise somewhat removed from such opportunities. Regular social media use can be beneficial to physically isolated Romanian providers who would benefit from their peers’ experiences in providing current HIV/AIDS practice standards in ways that would reduce HIV/AIDS stigma surrounding identification and treatment. These findings support the use of social media platforms to connect HIV/AIDS providers in Romania, which may also be applicable in other low-resource settings.

## Paper context

Social media is an innovative option for connecting clinicians together and enhancing access to professional resources. Romania has an atypical HIV/AIDS epidemic which is further challenged by a range of access complexities. Romanian HIV/AIDS providers used Facebook and WhatsApp most frequently, and those using one media platform were significantly more likely to use another, suggesting that social media could be used to create an online training platform on HIV/AIDS for Romanian clinical providers.
